# Adding Electroanatomical Mapping to Cryoballoon Pulmonary Vein Isolation Improves 1-Year Clinical Outcome and Durability of Pulmonary Vein Isolation: A Propensity Score-Matched Analysis

**DOI:** 10.3390/jcdd11020057

**Published:** 2024-02-06

**Authors:** Maxime Tijskens, Juan Pablo Abugattas, Hendrik Thoen, Antanas Strazdas, Bruno Schwagten, Michael Wolf, Yves De Greef

**Affiliations:** 1 Department of Cardiology, ZNA Heart Centre Middelheim, 2020 Antwerp, Belgiumbruno.schwagten@zna.be (B.S.); michael.wolf@zna.be (M.W.);; 2 AZ Rivierenland Hospital, 2840 Bornem, Belgium; 3 Heart Rhythm Management Centre, Postgraduate Program in Cardiac Electrophysiology and Pacing, Universitair Ziekenhuis Brussel-Vrije Universiteit Brussel, European Reference Networks Guard-Heart, 1050 Brussels, Belgium; 4 CHU UCL Namur, Site Sainte-Elisabeth, 5000 Namur, Belgium

**Keywords:** atrial fibrillation, pulmonary vein isolation, electroanatomical mapping, cryoballoon ablation, pulmonary vein reconnection

## Abstract

**Background:** Adding electroanatomical left atrial (LA) voltage mapping to cryoballoon ablation (CBA) improves validation of acute pulmonary vein isolation (PVI). **Aims:** To determine whether the addition of mapping can improve outcome and PVI durability. **Methods:** One-year outcome and PV reconnection (PVR) rate at first repeat ablation were studied in 400 AF patients in a propensity-matched analysis (age, AF type, CHA_2_DS_2_-VASc score) between Achieve catheter-guided CBA with additional EnSite LA voltage maps performed pre- and post-CBA (mapping group; N = 200) and CT- and Achieve catheter-guided CBA (control group; N = 200). Clinical success was defined as freedom of documented AF or atrial tachycardia (AT) > 30 s. PV reconnection patterns were characterized in repeat ablations. **Results:** At 1 year, 77 (19.25%) patients had recurrence of AF/AT, significantly lower than in the mapping group: 21 (10.5%) vs. 56 (28%), *p* < 0.001. Procedure time was shorter (72.2 ± 25.4 vs. 78.2 ± 29.3 min, *p* = 0.034) and radiation exposure lower (4465.0 ± 3454.6 Gy.cm^2^ vs. 5940.5 ± 4290.5 Gy.cm^2^, *p* = 0.037). Use of mapping was protective towards AF/AT recurrence (HR = 0.348; 95% CI 0.210–0.579; *p* < 0.001), independent of persistent AF type (HR = 1.723; 95% CI 1.034–2.872; *p* = 0.037), and LA diameter (HR = 1.055; 95% CI 1.015–1.096; *p* = 0.006). At repeat ablation (N = 90), persistent complete PVI was seen in 14/20 (70.0%) versus 23/70 (32.9%) in the mapping and conventional group, respectively (*p* = 0.03). Reconnection rate of the right inferior PV was lower with mapping (10.0% vs. 34,3%, *p* = 0.035). **Conclusions:** Adding electroanatomical LA voltage mapping to CBA improves 1-year clinical outcome and lowers both procedure time and radiation exposure. At repeat, use of mapping increases complete persistent PVI mainly by improving PVI durability of the RIPV.

## 1. Introduction

Cryoballoon ablation (CBA) is an established alternative to radiofrequency ablation (RFA) to perform pulmonary vein isolation (PVI) in symptomatic atrial fibrillation (AF) [1]. More recent evidence proved CBA superior to antiarrhythmic drugs as first-line therapy in paroxysmal AF for reducing atrial arrhythmia recurrence [2,3,4]. During recent years, our group and others could identify suboptimal PVI validation when using solely the 8-polar inner-lumen spiral mapping catheter (Achieve™, Medtronic Inc., Minneapolis, MN, USA) as an important limitation of CBA. Unrecognized incomplete PVI as a result can be an important cause of AF recurrence after CBA [5,6,7,8]. The recent Achieve Plus study confirmed that the Achieve catheter sometimes fails to detect incomplete PVI, leaving unrecognized non-isolated PVs in 12% of the patients [8]. Adding electroanatomical left atrial (LA) voltage mapping pre- and post-CBA was able to overcome this limitation improving the acute PVI rate compared to Achieve mapping only, however, at the expense of longer procedure times (±10 min) with no difference in fluoroscopy times [8]. The clinical impact of this more precise PVI validation is, although expected to be positive, still to be demonstrated. 

Hence, the present study investigated whether adding of electroanatomical LA voltage mapping to CBA can improve 1-year clinical outcome by comparison in a propensity-matched analysis of 200 patients undergoing CBA with mapping (Mapping group) versus 200 patients undergoing conventional CBA (Control group). Furthermore, durability of PVI at first repeat ablation studies was compared between both groups. 

## 2. Methods

### 2.1. Study Population

A total of 400 patients with symptomatic AF undergoing a first PVI with the cryoballoon at our institution (Middelheim Heart Center, Antwerp, Belgium) were compared in a propensity score-matched analysis between Achieve catheter-guided CBA with additional LA voltage maps performed pre- and post-CBA with the Achieve catheter and the EnSite™ cardiac mapping system (Abbott Inc., St. Paul, MN, USA) (Mapping group, N = 200) and standard fluoroscopy-only Achieve catheter-guided CBA (Control group, N = 200). The mapping group was ablated in the time period from January 2020 to October 2020. Propensity score-matching was performed in a 1:1 fashion with patients derived from the Middelheim-PVI registry 2 encompassing 1000 patients undergoing CBA from January 2017 to March 2019. Obviated in the mapping group, all control patients underwent contrast-enhanced CT-scan prior to ablation to establish the anatomy of the LA and PVs. AF was classified as ‘paroxysmal’ in case of spontaneous termination or with intervention within 7 days, or ‘persistent’ if sustained beyond 7 days according to the 2020 AF guidelines [9]. The study was approved by the local ethics committee.

### 2.2. Cryoballoon Ablation Procedure

The set-up of our CBA protocol has been previously described in detail [10]. In brief, after gaining access to the left atrium (LA), a 15-Fr steerable sheath (Flex Cath 18 Advance™, Medtronic Inc., Minneapolis, MN, USA) was advanced into the LA cavity, followed by a 28-mm CB (Arctic Front Advance™ or Arctic Front Advance Pro™, Medtronic Inc., Minneapolis, MN, USA) with a octapolar 20-mm diameter inner lumen spiral mapping catheter (Achieve™, Medtronic Inc., Minneapolis, MN, USA). Then the CB was advanced over the Achieve, inflated in the LA, and positioned at each PV ostium. Optimal vessel occlusion was confirmed by selective PV angiograms. Prior to ablation, all effort was made to record LA-PV potentials with the Achieve catheter (moving to a more proximal position, different torquing movements, etc.), allowing real-time monitoring of PV entrance block. Veins were ablated using a single freeze strategy per vein either 180 or 240 s. The choice for either of them was left to operator discretion. However, if at 60 s freeze time, pulmonary vein potential (PVP) did not disappear, and/or the temperature of −40 °C was not reached, cryoablation was either aborted and followed by catheter repositioning to attempt a better occlusion, or continued, and a second application was given in order to reach the target parameters mentioned above. The right phrenic nerve (PN) was monitored continuously during the ablation of the right-sided PVs. In case of transient phrenic nerve injury, no additional cryo-ablations were applied at the level of the right PVs. At the end of ablation, all PVs were re-checked in sinus rhythm with the Achieve catheter to confirm PVI defined as PV entrance block. Pacing maneuvers were used to distinguish residual PVPs from far-field signals (pacing from superior vena cava and distal coronary sinus for respectively right-sided and left-sides pulmonary veins). In case of residual PVPs, additional cryo-ablations were delivered until PVI was achieved. In case of a left common pulmonary vein (LCPV), the superior branch was defined as left superior pulmonary vein (LSPV), whereas the inferior branch was defined as the left inferior pulmonary vein (LIPV). Procedure-related complication was defined as any procedure-related adverse event occurring up to 1 month after ablation. 

### 2.3. Electroanatomical Voltage Mapping

In the Mapping group, additional pre-and post-electroanatomical LA voltage maps specifically mapping in detail the PVs, PV antral region and posterior wall, were performed using the Achieve catheter and EnSite™ cardiac mapping system (Abbott Inc., St. Paul, MN, USA). 

**Figure 1:**
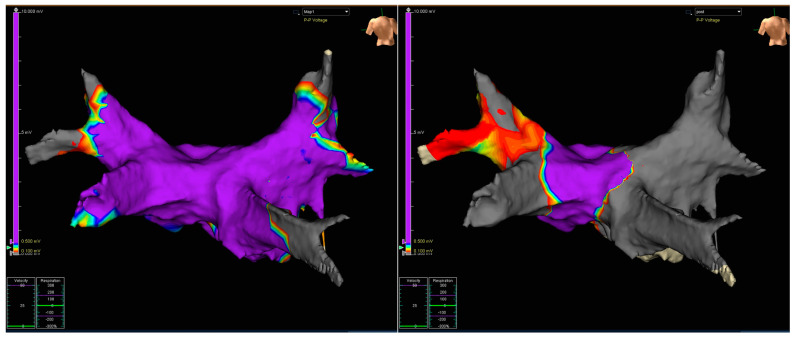
Electroanatomical voltage maps pre- and post-CBA in the Mapping group. **Left panel**: Pre-ablation normal voltaged left atrium with muscular sleeves extending into the 4 PVs on the baseline bipolar voltage map. **Right panel**: post-ablation bipolar voltage map with antral ablation tissue demarcation at the LA-PV junction. Cut-off values voltage map: >0.500 mV: purple; <0.100 mV: grey.

**Figure 2:**
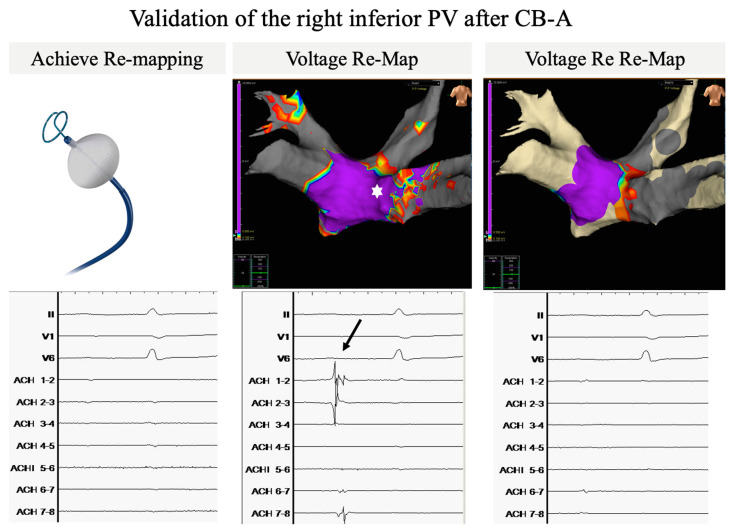
Representative example of a PVI validation of the right inferior PV in the Mapping group. **Lower panels**: surface ECG leads II/V1/V6 and bipolar electrogram recordings of the Achieve catheter positioned at the ostium of the right inferior PV are shown. Although Achieve EGM’s when re-mapping the RIPV after CBA show no residual PV signals (**Left panel**), a broad posterior LA-PV connecting tissue strain (marked by white asterisk, **Middle panel**) is still present on the validation map indicative of persistent LA-PV connection, further confirmed by clear PV signals (arrow) with the Achieve positioned at the site of the connecting sleeve. After additional CB-ablations PVI is confirmed by the disappearance of PV signals on Achieve EGM (**Right lower panel**) and the disappearance of the connecting LA-PV sleeve on the Ensite validation re-map (**Right upper panel**). Cut-off values voltage map: >0.500 mV: purple; <0.100 mV: grey. Abbreviations: PV: pulmonary vein; PVI: pulmonary vein isolation; CBA: cryoballoon ablation; LA: left atrial.

While in the Control group PVI was validated solely with the Achieve-PVP signal mapping, in the Mapping group validation of PVI was done by combined Achieve-PVP signal mapping and a second voltage map using the Achieve catheter and the Ensite system, allowing the comparison of the pre- and post-ablation voltage maps. Low-voltage areas were defined using the conventional cutoff < 0.5 mV, as reported in previous RFA studies [11,12]. Representative examples are shown in Figure 1 and Figure 2. Figure 1 shows the electroanatomical Achieve/Ensite LA voltage maps performed pre-and post-CBA in the Mapping group. 

Figure 2 illustrates how the extra validation step of electroanatomical mapping can overcome limitations of Achieve-only PVP signal validation. Although Achieve EGM’s when re-mapping the right inferior PV after CBA show no residual PV signals (left panel), a broad posterior LA-PV connecting tissue strain (marked by white asterisk, middle panel) is still present on the validation map indicative of persistent LA-PV connection, further confirmed by clear PV signals (arrow) with the Achieve positioned at the site of the connecting sleeve. After additional CB-ablations PVI is confirmed by the disappearance of PV signals on Achieve EGM (right lower panel) and the disappearance of the connecting LA-PV sleeve on the validation re-map (right upper panel). 

### 2.4. Post-Procedural Management and Follow-Up

After the procedure, subcutaneous low-molecular weight heparin was administered to all patients, as well as oral anticoagulation therapy (OAT), either a vitamin K antagonist (target INR between 2.0 and 3.0) or a direct oral anticoagulant. Antiarrhythmic drug treatment was reinstituted in all patients. After 2 months, OAT was continued except if a CHA2DS2-VASc score of 0 or 1 was observed in females. Whereas all antiarrhythmic drugs were invariably stopped, except for beta-blocking agents. 

All patients underwent 1-year follow-up with questionnaire, physical examination and electrocardiogram at scheduled visits (at 2, 6 and 12 months) and at unscheduled visits (if symptomatic). In the latter, the related arrhythmia was documented either by ECG, Holter monitoring (1 to 7 days), or event recording. The primary endpoint for this analysis was clinical success, defined as freedom from documented atrial arrhythmia (AF or atrial tachycardia) at least 30 s in duration after a single first procedure, as recommended by the Expert Consensus Statement of 2017 [13]. All first repeat ablation procedures during total follow-up (and not only repeat ablations of patients with a recurrence during the first year) were included for analysis.

### 2.5. Repeat Ablation Procedures 

All repeat procedures were performed using point-by-point RF ablation guided by electroanatomical mapping system (Carto, Biosense Webster, Diamond Bar, CA, USA) in combination with a multipolar mapping catheter (Lasso or Pentaray, Biosense Webster, Diamond Bar, CA, USA). At baseline, after creation of a left atrial voltage map, the PV status was checked and re-isolation was performed in case of PV reconnection. The additional strategy was left to the individual operator’s choice on a patient per patient basis and consisted of atrial pacing with and without the use of intravenous isoprenaline to assess the presence of non-PV triggers or inducible arrhythmias, scar-based ablation in the presence of scar, posterior box isolation, empiric creation of linear lines or empirical ablation of common non-PV triggers such as the superior vena cava.

## 3. Statistical Analysis 

Categorical variables are expressed as absolute and relative frequencies. The Shapiro-Wilk test was used to examine if a continuous variable was normally distributed. Continuous variables are expressed as mean ± standard deviation or median and range as appropriate. Event-free survival rates were estimated by the method of Kaplan–Meier. Comparisons of continuous variables were done with a Student’s t-test or Mann–Whitney U test as appropriate and comparisons of categorical variables with the χ^2^ or the Fisher’s exact test as appropriate. For each variable, hazard ratio (HR), 95% confidence interval (CI), and *p*-values of the final model are displayed. A two-tailed probability value of 0.05 was deemed significant. Statistical analyses were conducted using SPSS data-analytical software (SPSS v24, Chicago, IL, USA).

## 4. Propensity Score Matching

Propensity score matching was performed to compare the Mapping and Control group. A total of 1200 patients, 200 in the Mapping group and 1000 in in the Control group, were matched in a 1:1 ratio based on propensity scores. Propensity scores were calculated for each patient using multivariable logistic regression based upon the covariates: age, CHA_2_DS_2_-VASc score and AF type using calipers of width equal to 0.2 of the standard deviation of the logit of the propensity score. This resulted in two balanced groups of 200 patients each. IBM SPSS Version 24 and PS Match3.04R ( IBM Corp, Armonk, NY, USA) extension for SPSS were used for the calculations.

## 5. Results

### Clinical and Procedural Characteristics

Baseline characteristics and procedural variables are outlined in Table 1 and Table 2, respectively. No major differences were seen between groups except a lower prior use of antiarrhythmic drugs in the Mapping group (53.0 versus 67.5% in the Control group, *p* = 0.003). This difference was driven by a greater proportion of Class Ic antiarrhythmic drug intake in the Control group (41.5 versus 32.0% in the Mapping group, *p* = 0.049). 

Successful acute PVI could be obtained in all PVs. Total freeze durations, number of veins with PVP recording during freeze (real-time PVI) as well as isolation times and temperatures of individual veins did not differ between groups. Mean freeze durations tended to be shorter for the right PVs in comparison to the left PVs. Most probably this reflects the preferential choice of operators to opt for 180 or 240 s freeze duration for the right and left PVs respectively. 

Procedure times were shorter in the Mapping group (72.2 ± 25.4 min vs. 78.2 ± 29.3 min, *p* = 0.034). Also, radiation exposure was less in the Mapping group (4465.0 ± 3454.6 Gy.cm^2^ vs. 5940.5 ± 4290.5 Gy.cm^2^, *p* = 0.037). 

Complications are listed in Table 3. There were no significant differences in complications between groups. A total of 4 (1.0%) major and 22 (5.5%) minor complications occurred in 400 patients. No periprocedural deaths nor atrio-esophageal fistula occurred. The great majority of complications (24 or 92%) resolved completely after treatment and without permanent sequelae. One patient in the Control group suffered from stroke with permanent visual disturbance and one patient in the Mapping group had a phrenic nerve injury that persisted at 1-year follow-up. All other patients with phrenic nerve injury experienced complete resolution during waiting time (maximum of 20 min). Of note, in 15 of 16 cases (93.8%), phrenic nerve injury occurred during CBA of the right superior PV.

## 6. Clinical Success 

**Figure 3:**
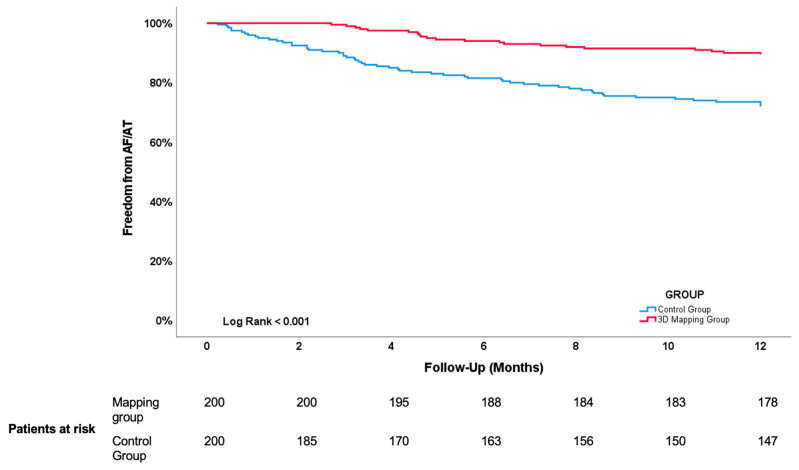
Kaplan–Meier analysis of atrial arrhythmia-free survival following CBA in patients with perprocedural electroanatomical mapping (Mapping group) and preprocedural CT scan (Control group). See text for further explanation.

**Figure 4:**
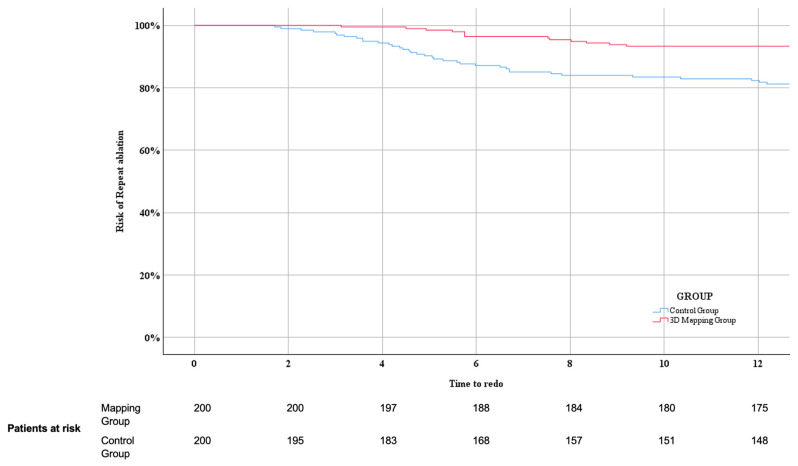
Kaplan–Meier estimates of undergoing a repeat ablation in patients with a recurrence within the first year of follow-up between Mapping and Control group. See text for further explanation.

Primary endpoint, defined as freedom of documented atrial arrhythmia at 1 year, was reached in 323 out of the 400 (80.8%) patients. As illustrated in Figure 3, the Kaplan–Meier estimate of clinical success was higher in the Mapping group: 179 (89.5%) remained free of AF versus 144 (72%) in the Control group (*p* < 0.001). Recurrent arrhythmia during first year of follow-up consisted of AF in 69 patients (18 in the Mapping group versus 51 in the Control group) and AT in 8 patients (3 in the Mapping group versus 5 in the Control group) (*p* = 0.150). Out of the 77 patients who suffered a recurrence within the first year, 49 (63.6%) patients underwent a repeat ablation: 13 (61.9%) in the Mapping group and 36 (64.3%) in the Control group (*p* = 0.850). The Kaplan–Meier estimate of undergoing a repeat ablation in patients with a recurrence within the first year of follow-up was significantly lower in the Mapping group (Log Rank 0.000), as shown in Figure 4. 

Predictors of AF recurrence are shown in Table 4, both in univariable- and multivariable-adjusted analyses. The use of electroanatomical LA voltage mapping was a powerful independent predictor of AF recurrence, both in univariate (hazard ratio (HR) 0.331; 95% confidence interval [CI] 0.200–0.547; *p* < 0.001) and multivariate analysis (HR 0.348; 95% CI 0.210–0.579; *p* < 0.001). Use of mapping was protective towards AF recurrence independent of persistent AF type (HR = 1.723; 95% CI 1.034–2.872; P=0.037), and LA diameter (HR = 1.055; 95% CI 1.015–1.096; *p* 0.006).

## 7. Repeat Ablation Findings 

A total of 90 patients underwent a first repeat ablation procedure: 20 patients in the Mapping group and 70 in the Control group. This difference in number of repeat ablation procedures has to be seen in a context of a much longer follow-up time in the Control group (25.6 ± 7.2 vs. 50.3 ± 3.3 months, *p* < 0.001). Time from index ablation until repeat ablation did not differ between the Mapping and Control group (18.0 ± 15.9 vs. 14.1 ± 11.4, *p* = 0.306).

Findings at repeat ablation are summarized in Table 5. 

**Figure 5:**
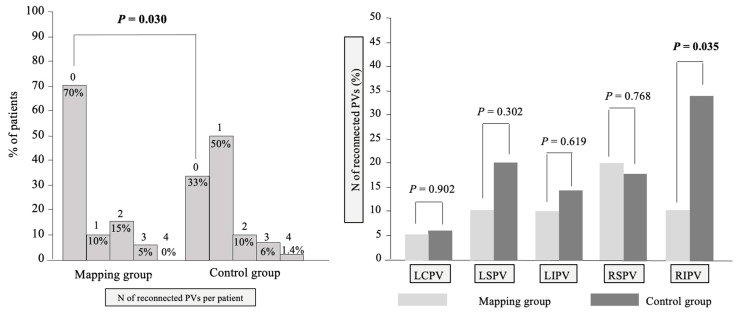
PV reconnection per patient and per PV at first repeat ablation study. **Left panel:** Graphical representation of the number of reconnected pulmonary veins per patient as seen during repeat ablation study. **Right panel:** Graphical representation of location of pulmonary vein reconnection per patient as seen during repeat ablation study. Abbreviations: LCPV, left common pulmonary vein; LSPV, left superior pulmonary vein; LIPV, left inferior pulmonary vein; RSPV, right superior pulmonary vein; RIPV, right inferior pulmonary vein.

The overall number of patients with PV reconnections was less in the Mapping group (6 out of 20 patients [30.0%] vs. 47 out of 70 patients [67.1%] in the Control group, *p* = 0.030) (Figure 5, left panel). The PV reconnection pattern per patient differed between groups (*p* = 0.018) with more patients with persistent complete PVI in the Mapping group (14 out 20 patients [70%] vs. 23 out of 70 (32.9%) in the Control group). On an individual PV level, less PV reconnections of the right inferior PVs were seen in the Mapping group (2 out of 20 patients [10.0%] vs. 24 out of 70 patients [34.3%], *p* = 0.035). There was no significant difference in reconnection rate of the other PVs (Figure 5, right panel). 

During the repeat ablation procedures 16 patients underwent an AT ablation, significantly more in the Mapping group: 8/20 patients versus 8/70 in the Control group (*p* = 0.004). The type of AT in the Mapping group consisted of 4 cavo-tricuspid isthmus-dependent atrial flutters, 3 mitral isthmus-dependent atrial flutters and 1 focal AT. Five CTI-dependent atrial flutters, two focal ATs and multiple atypical atrial flutters were the ablated ATs in the Control group.

## 8. Discussion 

### Main Findings

To the best of our knowledge, this study is the first to show that adding electroanatomical LA voltage mapping to CBA in comparison to conventional CBA:

1. Lowers atrial arrhythmia recurrence risk and risk to undergo repeat ablation at 12 months.
2. Shortens procedure time and lowers radiation dose exposure.
3. Improves the number of patients with persistence of complete PVI mainly driven by improving the durability of pulmonary vein isolation of the right inferior PV.

## 9. Improved 1-Year Outcome and Lower Risk to Undergo Repeat Ablation by Mapping

The clear superiority in 1-year clinical outcome after CBA-mapping procedures over conventional CBA (89.5 versus 72%, (*p* < 0.001)) and a lowered risk of undergoing a repeat ablation, proves for the first time a clinical benefit of adding voltage mapping to CBA. This is in contrast to previous studies on voltage mapping in CBA [6,14]. Meissner et al. compared short-term clinical follow-up between 24 patients who underwent CBA with additional mapping by the High-Density Mesh Mapper with a historical cohort of 167 patients [6]. There were no significant differences in freedom from atrial arrhythmia recurrence at 3 months (83 vs. 82%, *p* = 0.78) and 6 months (76 vs. 79%, *p* = 0.660). However, they did not provide any baseline or procedural characteristics to be able to compare groups [6]. Guhl et al. retrospectively compared 334 non-matched patients undergoing conventional CBA with 98 patients undergoing CBA with additional mapping (45 Ensite and 53 CARTO). There were no significant differences in 1-year atrial arrhythmia recurrence free rates between no mapping, Ensite and CARTO groups (64.6 vs. 65.0 vs. 64.9%, *p* = 0.278) [14]. However, there was a trend to more persistent AF in the mapping groups (20.7 vs. 24.4 vs. 35.8%), a known predictor of worse outcome [14]. 

## 10. Improved Acute PVI with Mapping 

PVI is the cornerstone of AF ablation, but durable electrical isolation of PVs remains challenging. Reconnection of previously isolated PVs has been regarded as the likely mechanism of AF recurrence after ablation [13]. Given the suboptimal capability of the Achieve catheter to detect real-time PV signals during CBA and as PVI validation tool, a proportion of AF recurrences after CBA could also be related to unrecognized incomplete acute PVI rather than to PV reconnection [5,6,7,8].

We consider a superior PVI validation, and hence superior acute PVI in the mapping group as the main explanation for the better clinical outcome in the mapping group. Mapping strengthens acute PVI by several factors. 

Firstly, it provides visualization of the Achieve catheter on the map and, in comparison to fluoroscopy, a more accurate delineation of the LA-PV junction enabling correct ostial positioning of the Achieve catheter when validating PVI after CBA. 

Secondly, it serves as an extra validation step on top of Achieve-PVP signal mapping. Residual LA-PV connecting tissue strains on the post-CBA map are a sign of incomplete achievement of PVI. Roving of the Achieve catheter to the site of the connecting LA-PV strain enabling tracing of residual PVPs is facilitated by mapping. After subsequent elimination of residual PVPs by extra ablations, re-mapping aids to confirm final isolation. 

Thirdly, electroanatomical voltage mapping could in particular have an added value in acute PVI validation of veins without real-time PVI recording. Recording of PVP with the Achieve catheter is not possible in every vein and still leaves approximately one out of 5 veins unrecorded despite newer generations CB (Arctic Front Advance Pro™) with the shortened catheter tip (reduced by 40%) combined with the redesigned Achieve advanced (25-mm loop size) mapping catheter and the use of torquing movements [15,16,17]. Recent data demonstrate that the absence of real-time recording of PVI by the Achieve catheter in any pulmonary vein during CBA of AF is a strong and independent predictor of AF recurrence [18]. Veins with no real-time PVI during CBA raise doubt on the achievement of acute PVI, therefore it is reasonable to assume that veins without real-time PVI harbor a higher risk of incomplete PVI than veins with real-time PVI. 

Fourthly, the finding of less PV reconnections at first repeat ablation in the Mapping group (30 vs. 67.1%, *p* = 0.030) driven by significantly less PV reconnections of the right inferior PVs (10.0 vs. 34.3%, *p* = 0.035) points to an important role of mapping, in particular for the right inferior PVs. Right inferior PVs are well known to be the most challenging veins to isolate and therefore harbor the highest risk of being left un-isolated. The right inferior PV is the vein with the lowest rate of real-time PVI recording due to shorter muscular sleeve length and reduced CB stability often requiring pushing the Achieve catheter more distally inside the vein. Anatomic variants with less axial angulation, their proximity to transseptal puncture site and perturbations induced by phrenic nerve pacing all lead to suboptimal balloon-tissue contact, further lowering the efficacy of the cryo-freeze. Unsurprisingly, right inferior veins are also the veins most prone to reconnections with the inferior part as a known predilection site [19]. 

## 11. Adding Electroanatomical Mapping Makes CBA Faster and Reduces Radiation Exposure

Interestingly, besides improving clinical outcome by offering a more precise PVI assessment, the integration of voltage mapping into CBA was associated with a reduction of procedure times by ≈6 min. Previous studies showed no difference or an increase in procedure time with the use of mapping during CBA refs. [6,8,14]. Meissner et al. showed a longer procedure time (127.5 ± 36.1 min) in 24 patients who underwent prior and subsequent to CBA additional mapping using the High-Density Mesh Mapper (BARD Electrophysiology, Lowell, MA, USA) [6]. Guhl et al. showed no difference in procedure time in patients undergoing conventional CBA as compared to patients undergoing CBA with additional electroanatomical mapping using Achieve catheter and Ensite mapping system (115.1 ± 28.0 min vs. 109.4 ± 34.0 min, *p* = 0.616), but a longer procedure time as compared to additional electroanatomical mapping using the Pentaray catheter and the CARTO system (167.8 ± 71.0 min, *p* < 0.001) [14]. 

Also, in the Achieve Plus study, procedure time in patients who underwent CBA with additional Ensite mapping procedures was +/− 10 min longer as compared to conventional CBA [8].

Potential explanations are the pre-CBA voltage map serving as a ‘road’ map leading to more accurate visualization of the PV’s and its side branches providing different options to the operator to optimize balloon positioning and stabilization at the PV ostium possibly leading to faster occlusions and hence shorter procedure times. In conventional CBA, a common difficulty is to estimate the position of the Achieve catheter only based on fluoroscopy mainly when re-checking each vein after CBA to confirm PVI. Not only can residual PVPs be missed by a too distal placement or suboptimal wall contact, it can also be challenging to differentiate between a real remaining PVP and a far-field LA potential, even with the use of differential pacing maneuvers. Mapping by providing visualization of the Achieve position on the map and clear differentiation of the LA-PV junction as mentioned above can help to solve this issue, and therefore can save time. This discrepancy in procedure time between the present study and the Achieve Plus study is striking given workflow was in essence similar in both studies using the Achieve catheter as mapping catheter generating Ensite voltage maps pre- and post-CBA. One potential explanation can be the difference in study design with the Achieve Plus study being a prospective study, while the current analysis is more a representation of real-life daily practice. Also, in contrast to Achieve Plus, an abbreviated mapping protocol was used in the current study focusing on the PV regions and posterior wall. Operator experience can also play a role. Integration of mapping in CBA is standard of care in our center since June 2019. Therefore, all operators are experienced using this technique as opposed to the Achieve Plus study where these techniques were not used commonly. 

The reduction of radiation in the electrophysiology lab is an important topic in modern practice and current guidelines suggest the implementation of electroanatomical mapping in several ablation procedures to reach this goal [20,21]. Guhl et al. reported a longer fluoroscopy time patients undergoing CBA with additional electroanatomical mapping using Achieve catheter and Ensite mapping system as compared to patients undergoing solely Achieve catheter guided CBA (31.3 ± 10.4 min vs. 25.9 ± 11.3 min; *p* = 0.015) [14]. However, we showed for the first time that addition of electroanatomical mapping to CBA reduces radiation exposure (4465.0 ± 3454.6 Gy.cm^2^ in the Mapping group vs. 5940.5 ± 4290.5 Gy.cm^2^ in the Control group; *p* = 0.037). Moreover, the total radiation exposure for the patient was further reduced by avoiding a preprocedural cardiac CT scan.

The current study adds further evidence to the safety of CBA. There were no periprocedural deaths or esophageal fistula and only a very small number of tamponade and stroke. Phrenic nerve injury is a classical complication of CBA but also occurred in a low number of patients with only 1 patient (0.25%) with persistent phrenic nerve injury at 1-year follow-up. Of note, in all but one patient, phrenic nerve injury occurred during cryoapplication of the RSPV. Since our ablation sequence ends with this PV, there was only one patient with non-targeted RSPV (patient with phrenic nerve injury occurring during during cryoapplication of the RIPV).

## 12. Clinical Implications and Future Directions

The clear clinical benefit further underbuilds routine implementation of voltage mapping into CBA. Until now, the requirement of a mapping system and extra multipolar mapping catheter were the main reasons hampering its routine clinical use. The workflow of using the Achieve catheter (already an integral part of the CBA system) as mapping catheter limits costs and complexity by avoiding the time-consuming process of switching to a separate mapping catheter. Given the added value of mapping, the additional cost seems justified, in our opinion, avoiding a higher number of AF recurrences and hence higher need for repeat ablations. 

Despite the favorable results presented in our work, we think that there exists margin for further improvements in the design of the Achieve catheter to augment its ability to detect PVPs and improve its mapping capabilities to deliver higher quality electroanatomical maps. 

The Achieve maps generated with the Ensite system suffice for PVI but the low-density (only 8 poles) and the spiral nature often prevent good wall contact, especially for the anterior LA, rendering the maps insufficient for reliable LA voltage information of fibrosis. 

Although not the subject of current study, the addition of electroanatomical mapping to CBA holds promise to facilitate segmental nonocclusive cryoballoon ablation of pulmonary veins and extrapulmonary vein targets such as the posterior wall or left atrial appendage [22]. 

The addition of electroanatomical mapping also holds promise in emerging technologies such as pulsed field ablation (PFA). Although not conventionally used in most available PFA catheters, Duytschaever et al. recently demonstrated its value in a novel mapping-integrated PFA system [23].

## 13. Limitations

Our study has some limitations. First, the study is retrospective and single-center with its inherent limitations, which may restrict our ability to draw substantial conclusions. Although propensity score-matching was performed to maximally compensate for possible bias, the clear beneficial impact on clinical outcome seen in the present study needs to be confirmed in a randomized controlled clinical trial. Second, only a subgroup of patients had cardiac implantable electronic devices and therefore, asymptomatic atrial arrhythmia episodes may have occurred unnoticed, leading to an overestimation of our success rate. Third, in roughly the same period periprocedural electroanatomical mapping was added to CBA as standard of care in our center, the transition from second to fourth generation cryoballoon catheter (shorter catheter tip facilitating real-time PVI) took place. Therefore, this switch might contribute to the observed improved outcome as reported by Manfrin et al. [24]. However, in contrast to their findings, we did not see a difference in real-time PVI between groups, pointing at the added effect of mapping independent of an improved PVI validation using the fourth generation cryoballoon. Finally, because Control group procedures were performed earlier than the Mapping group procedures, improved outcome due to increased operator experienced cannot completely be ruled out. However, this effect is expected to be very minor because the operators were already well-experienced with CBA and the timing of the control procedures.

## 14. Conclusions

The present study demonstrates that adding electroanatomical LA voltage mapping to CBA improves 1-year clinical outcome, shortens procedure time and lowers radiation exposure in comparison to conventional CBA. In patients undergoing a first repeat ablation, the use of mapping improved the number of patients with persistent complete PVI mainly driven by a more durable PVI of the right inferior PV. Given the beneficial clinical and procedural results, the proposed workflow in the study could facilitate routine use of mapping in CBA. 

## Figures and Tables

**Table 1:** Baseline characteristics.

	Total (N = 400)	Control Group (N = 200)	Mapping Group (N = 200)	*p*-Value
Age	64.0 ± 10.1	63.3 ± 10.0	64.7 ± 10.3	0.154
Female sex	152 (38.0%)	76 (38.0%)	76 (38.0%)	1.000
Length (cm)	173.8 ± 11.6	174.5 ± 9.3	173.1 ± 13.5	0.242
Weight (kg)	85.5 ± 18.1	85.0 ± 15.9	86.0 ± 20.1	0.591
BMI (kg/m^2^)	29.2 ± 8.3	27.9 ± 4.7	30.4 ± 11.2	0.172
Diagnosis-to-ablation time (months)	30.6 ± 31.4	33.2 ± 41.7	28.1 ± 15.1	0.107
Paroxysmal AF	212 (53.0%)	107 (53.5%)	115 (52.5%)	0.841
LA diameter (mm)	41.5 ± 6.4	42.0 ± 6.1	41.1 ± 6.5	0.141
Arterial hypertension	199 (49.8%)	90 (45.0%)	109 (54.5%)	0.057
Diabetes	49 (12.3%)	24 (12.0%)	25 (12.5%)	0.879
Previous stroke/TIA	31 (7.8%)	15 (7.5%)	16 (8.0%)	0.852
CHA_2_DS_2_-VASC score > 2 points	141 (35.3%)	66 (33.0%)	75 (37.5%)	0.346
Obstructive sleep apnea	42 (10.5%)	25 (12.5%)	17 (8.5%)	0.192
Obesity	115 (28.8%)	56 (28.0%)	59 (29.5%)	0.740
Endurance exercise	28 (7.0%)	14 (7.0%)	14 (7.0%)	1.000
Tachycardia-induced cardiomyopathy	46 (11.5%)	23 (11.5%)	23 (11.5%)	1.000
Prior use of anti-arrhythmic drugs	241 (60.3%)	135 (67.5%)	106 (53.0%)	**0.003**
Betablocker	296 (74.0%)	145 (72.5%)	151 (75.5%)	0.494
Class Ic	147 (36.8%)	83 (41.5%)	64 (32.0%)	**0.049**
Amiodarone	92 (23.0%)	41 (20.5%)	51 (25.5%)	0.235
Sotalol	43 (10.8%)	27 (13.5%)	16 (8.0%)	0.076

Data are presented as mean value ± SD or number (%). Abbreviations: BMI, body mass index; AF, atrial fibrillation; LA, left atrium; TIA, transient ischemic attack.

**Table 2:** Procedural characteristics.

	Total (N = 400)	Control Group (N = 200)	Mapping Group (N = 200)	*p*-Value
Procedure time (min)	75.3 ± 27.6	78.2 ± 29.3	72.2 ± 25.4	**0.034**
Dose area product (Gy.cm^2^)	4691.4 ± 3622.9	5940.5 ± 4290.5	4465.0 ± 3454.6	**0.037**
LSPV				
Total freeze duration (s)	214 ± 31.6	216.1 ± 31.0	211.9 ± 32.0	0.152
Time at 40 °C (s)	54.0 ± 23.5	51.7 ± 21.3	54.9 ± 24.4	0.240
Temperature at 60 s (°C)	−42.9 ± 5.1	−42.7 ± 4.5	−43.0 ± 5.4	0.575
Time to isolation (s) *	49.3 ± 27.4	52.9 ± 31.9	47.9 ± 25.5	0.309
Temperature at isolation (°C) *	−36.6 ± 8.2	−37.0 ± 7.5	−36.4 ± 8.4	0.628
Minimal temperature (°C)	−50.8 ± 6.4	−50.4 ± 5.7	−51.0 ± 6.7	0.417
PVP recording during freeze (N, %)	71.2%	74.6%	70.0%	0.469
LIPV				
Freeze duration (s)	214.7 ± 32.2	215.9 ± 29.5	213.5 ± 34.5	0.411
Time at 40 °C (s)	64.0 ± 29.6	64.0 ± 31.5	64.0 ± 28.9	0.996
Temperature at 60 s (°C)	−40.7 ± 4.9	−40.8 ± 3.7	−40.7 ± 5.3	0.783
Time to isolation (s) *	37.5 ± 23.7	38.0 ± 24.1	37.3 ± 23.7	0.858
Temperature at isolation (°C) *	−29.7 ± 10.5	−30.9 ± 8.5	−29.2 ± 11.1	0.300
Minimal temperature (°C)	−46.4 ± 5.9	−46.4 ± 4.6	−46.4 ± 6.3	0.998
PVP recording during freeze (N, %)	67.8%	63.6%	69.3%	0.398
RSPV				
Freeze duration (s)	182.1 ± 31.3	180.2 ± 12.0	183.9 ± 41.7	0.203
Time at 40 °C (s)	48.2 ± 20.6	45.2 ± 15.1	49.3 ± 22.3	0.067
Temperature at 60 s (°C)	−44.0 ± 4.6	−44.7 ± 4.1	−43.7 ± 4.8	0.071
Time to isolation (s) *	34.5 ± 21.2	37.0 ± 20.0	33.6 ± 21.6	0.292
Temperature at isolation (°C) *	−30.5 ± 10.3	−32.7 ± 9.6	−29.6 ± 10.4	0.052
Minimal temperature (°C)	−51.1 ± 5.6	−51.9 ± 4.7	−50.8 ± 5.9	0.105
PVP recording during freeze (N, %)	72.5%	73.3%	72.2%	0.854
RIPV				
Freeze duration (s)	203.3 ± 37.6	206.0 ± 35.3	200.9 ± 39.5	0.145
Time at 40 °C (s)	61.5 ± 30.9	60.7 ± 33.7	61.8 ± 29.8	0.780
Temperature at 60 s (°C)	−41.4 ± 5.4	−41.9 ± 4.4	−41.2 ± 5.7	0.234
Time to isolation (s) *	42.5 ± 31.1	41.9 ± 34.0	42.7 ± 30.0	0.891
Temperature at isolation (°C) *	−32.1 ± 9.6	−31.7 ± 9.9	−32.2 ± 9.6	0.761
Minimal temperature (°C)	−48.7 ± 6.5	−48.9 ± 5.8	−48.6 ± 6.7	0.653
PVP recording during freeze (N, %)	64.7%	65.3%	64.5%	0.898

* In patients with real-time PVI. Data are presented as the mean value ± SD or number (%) of patients. Abbreviations: LSPV, left superior pulmonary vein; LIPV, left inferior pulmonary vein; RSPV, right superior pulmonary vein; RIPV, right inferior pulmonary vein; PV, pulmonary vein. PVP, pulmonary vein potential.

**Table 3:** Procedural complications.

	Total (N = 400)	Control Group (N = 200)	Mapping Group (N = 200)	*p*-Value
Major complications	4 (1.0%)	2 (1.0%)	2 (1.0%)	1.000
Cardiac tamponade	1 (0.3%)	1 (0.5%)	0 (0%)	0.317
Severe bleeding	0 (0%)	0 (0%)	0 (0%)	1.000
Persistent phrenic nerve injury	1 (0.3%)	0 (0%)	1 (0.5%)	0.317
Stroke/transient ischemic attack	2 (0.5%)	1 (0.5%)	1 (0.5%)	1.000
Esophageal fistula	0 (0%)	0 (0%)	0 (0%)	1.000
Death	0 (0%)	0 (0%)	0 (0%)	1.000
Minor complications	22 (5.5%)	13 (6.5%)	9 (4.5%)	0.380
Minor bleeding	0 (0%)	0 (0%)	0 (0%)	1.000
Pericarditis	5 (1.3%)	3 (1.5%)	2 (1.0%)	0.653
Transient air embolism	2 (0.5%)	1 (0.5%)	1 (0.5%)	1.000
Aneurysma spurium	0 (0%)	0 (0%)	0 (0%)	1.000
Transient phrenic nerve injury	15 (3.8%)	9 (4.5%)	6 (3.0%)	0.430

Data are presented as the number (%) of patients.

**Table 4:** Univariate and multivariate analysis of variables associated with atrial arrhythmia recurrence during follow-up.

	Univariate Analysis			Multivariate Analysis		
	HR	95% CI	*p*-Value	HR	95% CI	*p*-Value
Diagnosis-to-ablation time (months)	1.001	0.994–1.008	0.766			
Age	1.028	1.003–1.053	0.027	1.010	0.978–1.044	0.548
Arterial hypertension	1.393	0.887–2.187	0.150			
BMI (kg/m^2^)	0.999	0.986–1.012	0.901			
CHA_2_DS_2_-VASC score	1.167	1.028–1.326	0.017	1.079	0.906–1.285	0.396
Female sex	1.108	0.703–1.748	0.658			
Perprocedural electroanatomical mapping	0.331	0.200–0.547	<0.001	0.348	0.210–0.579	**<0.001**
LA diameter	1.088	1.050–1.128	<0.001	1.055	1.015–1.096	**0.006**
Obstructive sleep apnea	1.875	1.033–3.405	0.039	1.665	0.914–3.033	0.096
Persistent AF type	2.269	1.421–3.624	0.001	1.723	1.034–2.872	**0.037**

Abbreviations: AF, atrial fibrillation, LA, left atrium.

**Table 5:** Spatial distribution of PV reconnection on a patient and PV level.

	Total (N = 90)	Control Group (N = 70)	Mapping Group (N = 20)	*p*-Value
Number of PVs reconnected per patient				
4	1 (1.1%)	1 (1.4%)	0 (0%)	**0.018**
3	5 (5.6%)	4 (5.7%)	1 (5.0%)	
2	10 (11.1%)	7 (10.0%)	3 (15.0%)	
1	37 (41.1%)	35 (50.0%)	2 (10.0%)	
0	37 (41.1%)	23 (32.9%)	14 (70.0%)	
PV Reconnection				
LCPV	5 (5.6%)	4 (5.7%)	1 (5.0%)	0.902
LSPV	16 (17.8%)	14 (20.0%)	2 (10.0%)	0.302
LIPV	12 (13.3%)	10 (14.3%)	2 (10.0%)	0.619
RSPV	16 (17.8%)	12 (17.1%)	4 (20.0%)	0.768
RIPV	26 (28.9%)	24 (34.3%)	2 (10.0%)	**0.035**
RMPV	0 (0%)	0 (0%)	0 (0%)	1.000

Data are presented as number (%). Abbreviations: LCPV, left common pulmonary vein; LSPV, left superior pulmonary vein; LIPV, left inferior pulmonary vein; RSPV, right superior pulmonary vein; RIPV, right inferior pulmonary vein; RMPV, right middle pulmonary vein.

## Data Availability

Data can be provided upon request.
